# The Controversial Role of 24-S-Hydroxycholesterol in Alzheimer’s Disease

**DOI:** 10.3390/antiox10050740

**Published:** 2021-05-07

**Authors:** Paola Gamba, Serena Giannelli, Erica Staurenghi, Gabriella Testa, Barbara Sottero, Fiorella Biasi, Giuseppe Poli, Gabriella Leonarduzzi

**Affiliations:** Department of Clinical and Biological Sciences, University of Turin, 10043 Turin, Italy; serena.giannelli@unito.it (S.G.); erica.staurenghi@unito.it (E.S.); gabriella.testa@unito.it (G.T.); barbara.sottero@unito.it (B.S.); fiorella.biasi@unito.it (F.B.); giuseppe.poli@unito.it (G.P.); gabriella.leonarduzzi@unito.it (G.L.)

**Keywords:** 24-S-hydroxycholesterol, cerebrosterol, oxysterol, brain cholesterol metabolism, Alzheimer’s disease, neuroprotection, neurodegeneration, CYP46A1, statins

## Abstract

The development of Alzheimer’s disease (AD) is influenced by several events, among which the dysregulation of cholesterol metabolism in the brain plays a major role. Maintenance of brain cholesterol homeostasis is essential for neuronal functioning and brain development. To maintain the steady-state level, excess brain cholesterol is converted into the more hydrophilic metabolite 24-S-hydroxycholesterol (24-OHC), also called cerebrosterol, by the neuron-specific enzyme CYP46A1. A growing bulk of evidence suggests that cholesterol oxidation products, named oxysterols, are the link connecting altered cholesterol metabolism to AD. It has been shown that the levels of some oxysterols, including 27-hydroxycholesterol, 7β-hydroxycholesterol and 7-ketocholesterol, significantly increase in AD brains contributing to disease progression. In contrast, 24-OHC levels decrease, likely due to neuronal loss. Among the different brain oxysterols, 24-OHC is certainly the one whose role is most controversial. It is the dominant oxysterol in the brain and evidence shows that it represents a signaling molecule of great importance for brain function. However, numerous studies highlighted the potential role of 24-OHC in favoring AD development, since it promotes neuroinflammation, amyloid β (Aβ) peptide production, oxidative stress and cell death. In parallel, 24-OHC has been shown to exert several beneficial effects against AD progression, such as preventing tau hyperphosphorylation and Aβ production. In this review we focus on the current knowledge of the controversial role of 24-OHC in AD pathogenesis, reporting a detailed overview of the findings about its levels in different AD biological samples and its noxious or neuroprotective effects in the brain. Given the relevant role of 24-OHC in AD pathophysiology, its targeting could be useful for disease prevention or slowing down its progression.

## 1. Introduction

Alzheimer’s disease (AD) is an unsolved health burden that accompanies increased life expectancy and is characterized by progressive memory destruction and alteration of other important brain functions.

In the past, a clinical diagnosis was used to identify probable cases of AD. The definitive diagnosis could only be confirmed post-mortem by identifying the main AD hallmarks which are the extracellular accumulation of amyloid-β (Aβ) peptides and the hyperphosphorylation of intracellular tau protein leading to senile plaque and neurofibrillary tangle (NFT) formation, respectively, in the brain [1,2]. More recently, several guidelines indicate the quantification of Aβ_42_, total tau (t-tau) and tau phosphorylated at threonine 181 (p-tau) in blood samples and in the cerebrospinal fluid (CSF) as indicators for AD clinical diagnosis [3,4,5,6,7].

Considerable evidence indicates that several events contribute to AD progression, including oxidative stress and neuroinflammation. Of note, it has been widely reported that increased oxidative stress in the AD brain intensifies neurodegeneration by favoring generation of reactive oxygen species (ROS) and lipid peroxidation [8,9]. At the same time, AD is associated with the dysregulation of cholesterol homeostasis in the brain, and hypercholesterolemia is included among risk factors. Maintenance of cholesterol homeostasis in the brain is essential for neuronal functioning and brain development. Since blood cholesterol cannot cross the blood brain barrier (BBB), in the adult brain most cholesterol derives from de novo synthesis that occurs mainly in astrocytes and, to a lesser extent, in neurons [10]. The synthesized cholesterol combines with apolipoprotein E (ApoE), produced by astrocytes, to form lipoproteins secreted into the extracellular fluid through ATP-binding cassette (ABC) transporters present on astrocyte cell membranes, and then transported to neurons [11,12]. ApoE-containing lipoproteins are taken up by two functionally important low-density lipoprotein (LDL) receptors: the prototypic LDL receptor (LDLR) and the LDL receptor-related protein 1 (LRP1). Although both are present in astrocytes and neurons, the LDLR is highly expressed in astrocytes whereas LRP1 is mainly expressed in neurons [13]. Following receptor-mediated endocytosis, ApoE is recycled to the plasma membrane, and cholesterol is used for cell membrane turnover and repair, myelin formation, synaptogenesis and neurotransmitter release [14,15,16].

To maintain the steady-state level, excess cholesterol is metabolized through three different pathways: (i) esterification and subsequent intracellular storage in lipid droplets, (ii) direct excretion via ABC transporters, (iii) conversion into the oxysterol 24-S-hydroxycholesterol (24-OHC).

**Figure 1:**
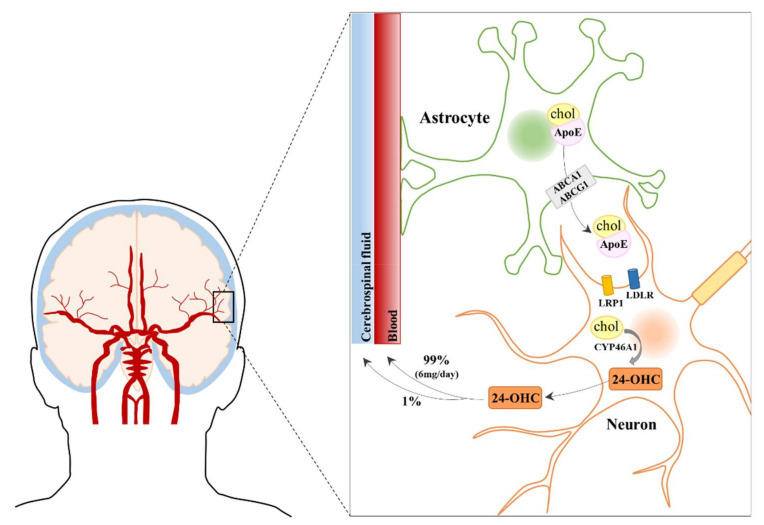
Fluxes of 24-S-hydroxycholesterol from the brain to the blood and the cerebrospinal fluid.

Concerning the last pathway, to maintain cholesterol homeostasis, cholesterol is converted into the more hydrophilic metabolite 24-OHC, also called cerebrosterol, by the neuron-specific enzyme CYP46A1, which is responsible for at least 40% of brain cholesterol conversion. This enzyme is highly expressed by certain types of neurons in the brain, such as pyramidal cells of the cortex and Purkinje cells of the cerebellum, making these cells particularly sensitive to excess cholesterol [17,18]. A great amount of the total 24-OHC in the body (80%) is present and produced in the brain [19,20], where its levels directly correlate to cholesterol levels. The majority of 24-OHC diffuses across the BBB into the systemic circulation driven by the concentration gradient and is then delivered to the liver for further degradation to bile acids [19,21,22,23]. It is estimated that approximately 1% of 24-OHC synthesized in the brain enters the CSF [22,24] (Figure 1). Furthermore, 24-OHC can be caught by astrocytes and neurons, where it up-regulate genes involved in cholesterol efflux [10].

To a lesser extent, cholesterol in the brain is also oxidized to 27-hydroxycholesterol (27-OHC) by the sterol 27-hydroxylase (CYP27A1), which is slightly expressed in neurons, astrocytes and oligodendrocytes, and then into 7α-hydroxy-3-oxo-4-cholestenoic acid (7-OH-4-C) by the oxysterol 7-alpha-hydroxylase (CYP7B1) [18,19,25]. Moreover, an inflow of extra-cerebral 27-OHC can also occur since this oxysterol is a major cholesterol metabolite in circulation and the 27-hydroxylase is ubiquitously expressed in the body. Overall, in physiologic conditions, there is an efflux of 24-OHC from the brain to the peripheral circulation, as well as an ingress of 27-OHC [26]. In the brain, homeostasis of the two oxysterols is tightly regulated in order to remain constant and specific for the different cerebral areas. For example, the 27-OHC:24-OHC ratio is 1:8 in the frontal cortex, 1:5 in the occipital cortex and 1:10 in the basal ganglia [27]. The oxysterol 7β-hydroxycholesterol (7β-OHC) also derives from cholesterol oxidation in the brain, following its interaction with amyloid precursor protein (APP) and Aβ [28]. Besides these, other oxysterols can be exported from the brain in the systemic circulation, including 7-ketocholesterol (7-KC) and 6-oxo-5α-hydroxycholesterol [20]. Two other cholesterol metabolites, 7α,25-dihydroxycholest-4-en-3-one and 7α,(25R)26-hydroxycholest-4-en-3-one, were reported to be exported from the brain [29].

A growing bulk of evidence suggests that oxysterols are the link connecting altered cholesterol metabolism to AD [10,30,31]. Toxic amounts of oxysterols can accumulate in the brain, particularly due to the increased flux of these sterol molecules from the peripheral circulation into the brain owing to increased permeability of the BBB [32]. Aging leads to partial disruption of the BBB integrity, but the barrier’s function can also be significantly affected in neurodegenerative diseases, including AD. Hypercholesterolemia associated with oxidative stress is considered one of the causes of this damage [19]. Furthermore, BBB integrity and function can be partially damaged by oxysterols themselves [33].

Oxysterols accumulating in the brain certainly play a crucial role in AD development by enhancing oxidative stress and inflammation, with consequent neurodegeneration [10]. Of note, although most oxysterols cause neuron dysfunction and degeneration, some have been recently shown to have neuroprotective effects. In particular, data about the role of 24-OHC in AD etiopathology are contrasting since they indicate either damaging or protective activities of this oxysterol. Paradoxically, even though 24-OHC is essential for the physiological elimination of excess cholesterol, it can also exert adverse effects. Such variability likely depends on the experimental model adopted, whose parameters (e.g., concentration of 24-OHC, cell types or animal species) can differently affect the outcome of the investigation and can be representative of different stages in disease progression.

In this review we summarized the current knowledge on the physiological role of 24-OHC in the brain and on its involvement in AD pathogenesis. In particular, we report a detailed overview of the findings published about its controversial effects on the brain, focusing on the different trends of its levels found in AD biological samples and on its noxious and beneficial effects in vitro.

## 2. The Physiological Role of 24-OHC in the Brain

The dominant oxysterol in the brain is 24-OHC and evidence shows that it represents a signaling molecule of great importance for brain function. Like other side-chain oxysterols, 24-OHC may favor membrane cholesterol accessibility, thereby altering membrane structure and indirectly influencing neuronal excitability [34].

The membrane biophysical properties of 24-OHC likely account for its ability in modulating cholesterol homeostasis [35]. This oxysterol is a physiological ligand of the transcription factors liver X receptors α (LXRα) and β (LXRβ) [36,37], and by this mechanism, 24-OHC acts as a physiological suppressor of brain cholesterol biosynthesis, mainly in astrocytes. 24-OHC also reduces cholesterol synthesis through LXR activation and subsequent inhibition of the sterol regulatory element binding protein (SREBP), which was recently observed in glioblastoma cells [38]. LXR activation by 24-OHC is also responsible for the expression and synthesis of ApoE and ABCA1/ABCG1 in astrocytes, which favor cholesterol transport from astrocytes to neurons [39,40].

Moreover, 24-OHC exerts a key role in maintaining cholesterol homeostasis in the neurovascular unit favoring cholesterol efflux. Indeed, it has been shown that 24-OHC increases expression of ABCA1 and ABCG1 in porcine [41,42] and ovine primary brain capillary endothelial cells (ECs) [43], as well as in bovine brain pericytes [44].

Notably, the oxysterol 24-OHC can also affect cholesterol homeostasis in neurons. Proteomic studies demonstrated that, in primary cortical neurons derived from embryonic rats, 24-OHC up-regulates ApoE expression likely via LXR. However, it also down-regulates the expression of enzymes in the cholesterogenic pathway in a post-transcriptional manner, by directly preventing the maturation of SREBP-1a and SREBP-2 transcription factors [45]. In human neuroblastoma SH-SY5Y cells, both SREBP-1 and SREBP-2 processing was prevented by 24-OHC. Of note, in the same cells, SREBP-1 gene expression and synthesis were up-regulated by the oxysterol via LXR activation, while SREBP-2 was down-regulated by an LXR-independent pathway [46].

24-OHC is also a ligand of retinoic acid receptor-related orphan receptors (RORs) [47]. It has been found to be an inverse agonist of RORα and RORγ, thus suppressing the constitutive activity of these receptors [48]. Of note, RORα, which is abundant in the cerebellum and thalamus, plays a key role in the development of Purkinje cells, where CYP46A1 is highly expressed [49], mainly affecting their maturation and survival [50]. Therefore, 24-OHC could play a key role in regulating maturation and survival of Purkinje cells via its inverse agonist activity towards RORα, although defects in cerebellum have not been observed in CYP46A1 knockout mice [51] or in mice overexpressing human CYP46A1 [52]. However, severe defects in motor learning have been observed in mice lacking CYP46A1 since their brain excretes cholesterol more slowly, and the tissue compensates by suppressing cholesterol synthesis [51].

Accumulating evidence also supports a major role of 24-OHC as a positive allosteric modulator of *N*-methyl-D-aspartate receptors (NMDARs), which mediate excitatory neurotransmission throughout the central nervous system (CNS) and are crucial for synaptic plasticity and learning [53,54,55].

## 3. 24-OHC Levels in Alzheimer’s Disease Biological Samples

Variable levels of 24-OHC were found in biological samples (brain, blood and CSF) from subjects with different AD severity. The subjects considered in the different studies were both male and female patients (female majority) between the ages of 55 to 85 years, and they were not suffering from chronic diseases, such as renal and hepatic dysfunction, diabetes mellitus or cancer. In most studies patients treated with cholesterol-lowering drugs and patients with alcohol abuse and dependence were excluded. Unfortunately, mean post-mortem intervals were not reported, although they are necessary to validate the results (Table 1).

As highlighted in Table 1, opposite trends in 24-OHC levels were often found in the same biological samples, indicating that it cannot yet be considered a reliable disease marker. Thus, further in vivo studies are needed to understand whether 24-OHC levels rise or fall as the disease progresses.

### 3.1. 24-OHC Levels in the Brain

Changes in the levels of 24-OHC in the brain reflect neuronal dysfunction during AD development. It has been demonstrated that, in critical areas of post-mortem brains of AD patients (frontal and occipital cortex, basal ganglia and pons), as well as in aged mice expressing the Swedish Alzheimer mutation APP751, 24-OHC levels decreased [27]. Similarly, Hascalovici and collaborators demonstrated that cerebral 24-OHC levels decreased with aging in the frontal cortex of AD individuals [56]. Moreover, a systematic analysis of oxysterols in autopsy specimens from the frontal and occipital cortex of human AD brains, classified as “early” or “late” AD based on the Braak staging system of neurofibrillary pathology, revealed that 24-OHC content significantly decreased in late AD compared to control and early AD brains. Notably, when all data regarding AD brains were grouped together without considering the disease stage of the donor, the reduction of 24-OHC levels was still considerable, but less significant [57]. On the other hand, 24-OHC levels increased in the frontal cortex in relation to age in individuals with mild cognitive impairment (MCI) or without cognitive impairment [56].

Therefore, considering these few data, one can presume that in the initial phases of the disease or with aging, 24-OHC levels do not change significantly, and they might even increase as a consequence of ongoing active neuronal destruction with increased liberation of total free sterols. However, in the more advanced stages of AD, 24-OHC markedly declines due to a selective loss of neurons expressing the enzyme CYP46A1. The decrease in both levels and activity of CYP46A1 could explain the observed decrease of 24-OHC in AD brains in later disease stages. In fact, in agreement with the trend of 24-OHC levels, CYP46A1 expression was also found to dramatically decrease in the frontal and occipital cortex of the AD brain during disease progression [57]. This decrement is associated with the decreased neuronal mass characteristic of the advanced stages of the disease [58].

### 3.2. 24-OHC Levels in Plasma

The levels of 24-OHC have been shown to change both in plasma and in the CSF during AD progression. More than 90% of the daily production of 24-OHC in the brain enters circulation via the BBB and by this way it is delivered to the liver, where it is further metabolized [22,24]. Therefore, 24-OHC plasma levels may be indicative of cholesterol homeostasis in the brain [21,59,60]. In adults, in the absence of neurodegeneration and liver disease, plasma 24-OHC is low and relatively stable until the sixth decade and then it declines with aging [61]. In the presence of BBB disturbance, however, alteration in 24-OHC plasma levels can occur. For example, in a mouse model with a defective BBB an increased leakage of 24-OHC out of the brain has been observed [62].

Plasma 24-OHC levels could also change with AD progression. After its initial elevation due to increased brain cholesterol turnover and increased flow through the damaged BBB, plasma levels drastically decrease in terminal stages as a consequence of the extensive loss of CYP46A1-expressing neurons [58,63,64,65]. It is worth noting that contrasting results have emerged, probably due to sample heterogeneity regarding the different stages of the disease. 24-OHC has been reported to increase [66,67] or decrease [68] in the blood of MCI or AD patients. Other studies on patients with early MCI or AD observed no significant changes in plasma 24-OHC compared to healthy control subjects [69,70].

A relationship between AD progression and 24-OHC, as well as other plasma lipids (HDL, LDL, total serum cholesterol, 27-OHC, triglycerides, lipoprotein A, phospholipids, and sphingolipids), has been observed [71]. This evidence supports the association between hypercholesterolemia/dyslipidemia and increased plasma levels of 24-OHC. For this reason, 24-OHC corrected for plasma cholesterol levels could be more informative [72].

The ratio of 24-OHC to total circulating cholesterol was found to be significantly lower in AD and MCI compared to controls [73]. Plasma 24-OHC/cholesterol was also analyzed in relation to grey matter and parenchymal volumes in subjects with AD, MCI or subjective cognitive impairment (SCI). The lowest ratio was in the AD group, the highest in the SCI group and an intermediate ratio was found in the MCI group. In all patients no significant correlation emerged between its value and brain volumes [74]. This trend is also suggested by the work of Papassotiropoulos and collaborators, where, after an initial increase in the earlier phases, reduction of the plasma 24-OHC/cholesterol was associated with severity of AD [75]. Lower levels of 24-OHC were detected in the serum of patients with greater AD severity associated with increased agitation, bringing out a decrease in 24-OHC levels in the advanced stages of the disease compared to controls [76,77]. A negative correlation between plasma 24-OHC levels and AD severity was found in another investigation, although in this case 24-OHC levels were higher in AD patients than in healthy controls [78]. A subsequent study partially confirmed this evidence, reporting increased plasma levels of 24-OHC in the early stage of late-onset AD patients compared to controls [79]. In contrast, plasma 24-OHC content was found to be lower in probable AD patients compared to controls, but this reduction did not reflect the clinical severity of the disease [80].

The reliability of assessing 24-OHC in blood when investigating AD is further questioned by the fact that no strong correlation was found between plasma 24-OHC and traditional AD biomarkers (i.e., Aβ, t-tau and p-tau) [81].

### 3.3. 24-OHC Levels in the Cerebrospinal Fluid

With regard to the possibility of using CSF as an alternative specimen for 24-OHC characterization in relation to AD, a positive correlation between 24-OHC concentrations and other AD-related species such as soluble APPα and β (sAPPα and sAPPβ) and tau protein levels has been identified in CSF samples of MCI and AD subjects [66]. The amount of 24-OHC was found to be higher in patients with BBB and blood-CSF barrier destruction. Of note, the small fraction of brain-derived 24-OHC in the CSF appears to reflect neuronal damage and is more consistently related to dementia than its amount in the plasma, where it could be strongly affected by hepatic clearance [32].

In the CSF of AD patients, higher concentrations of 24-OHC were observed due to increased cholesterol turnover during neurodegeneration, both at early and advanced stages compared to control-like subjects [82,83,84,85,86,87]. However, Griffiths and collaborators found no statistical differences in CSF concentrations of unesterified 24-OHC between AD and control groups [88], and other papers indicate reduced levels of 24-OHC in the CSF of AD subjects [89,90,91]. Of note, specific CYP46A1 gene variants have been recognized as risk factors for AD by influencing brain cholesterol metabolism and are responsible for reduced CSF levels of 24-OHC [91]. Lower levels of 24-OHC have been found in the CSF and in the plasma of AD patients carrying the retinoic X receptor α (RXRα) polymorphism rs3132293. The nuclear hormone receptors RXRs are key regulators of cholesterol synthesis and metabolism and RXRα gene variants might act as a risk factor for AD by influencing cerebral cholesterol metabolism [90].

**Table 1:** Changes in 24-OHC levels in the brain, blood and cerebrospinal fluid of MCI or AD subjects.

	Brain	Plasma	Cerebrospinal Fluid
**↑ levels of 24-OHC**	Post-mortem human MCI brain (frontal cortex) with aging [56]	MCI, AD versus control subjects [67]	MCI, AD versus control subjects [66,67]
		AD versus control subjects [66,78,79]	AD versus control subjects [82,83,84,85,86,87]
↓ levels of 24-OHC or 24-OHC/chol *	Post-mortem human AD brain (frontal and occipital cortex, basal ganglia, pons) versus control subjects [27]	AD versus control subjects [68,76,77,80]	AD subjects genotyping for RXRα polymorphism versus control subjects [90]
	Post-mortem human AD brain (frontal cortex) with aging [56]	AD subjects genotyping for RXRα polymorphism versus control subjects [90]	AD subjects genotyping for CYP46A1 polymorphism versus control subjects [91]
	Post-mortem human AD brain (frontal and occipital cortex) in later stages [57]	MCI, AD versus control subjects [73] *	
		MCI, AD versus SCI subjects [74] *	
		AD subjects with AD progression [75] *	
**No differences in 24-OHC levels**		MCI, AD versus control subjects [69]	AD versus control subjects [88]
		MCI versus control subjects [70]	

* papers which report 24-OHC/cholesterol ratio. Abbreviations: AD: Alzheimer’s disease; chol: cholesterol; CYP46A1: cholesterol 24-hydroxylase; 24-OHC: 24-S-hydroxycholesterol; MCI: mild cognitive impairment; RXRα: retinoid X receptor α; SCI: subjective cognitive impairment.

## 4. The Role of 24-OHC in Alzheimer’s Disease

It is now well accepted that during AD development certain oxysterols accumulating in the brain can act as friends and/or foes [92]. Among the different oxysterols, 24-OHC certainly has the most controversial role. On the one hand, it promotes neuroinflammation, Aβ peptide production, oxidative stress and cell death in neuronal cell lines [10,93,94,95,96,97]. On the other hand, 24-OHC has been reported to be a main player of the regulatory loop between astrocytes and neurons to maintain brain cholesterol homeostasis, and to exert several beneficial effects against AD progression, such as preventing tau hyperphosphorylation [98], suppressing Aβ production [99] in neuroblastoma cells and regulating synaptic function in rat hippocampal neurons and slices [54].

The different effects exerted by 24-OHC appear to depend on its concentration. In fact, high concentrations of 24-OHC (25–50 µM) are toxic to neuroblastoma SH-SY5Y cells [95], while low sub-lethal concentrations of 24-OHC (1–10 µM) within the range observed in the human brain induce an adaptive and neuroprotective response. This occurs via activation of LXRs [100], transcription factors that regulate cholesterol elimination, fatty acid and triglyceride biosynthesis, glucose metabolism and immune-inflammatory responses [101]. It displays different effects depending on its levels on human glioblastoma U-87 MG cells, where low concentrations (1–5 µM) of 24-OHC stimulate cellular processes critical to maintain redox homeostasis, while higher doses (10–20 µM) increase lipid and protein oxidative damage [102].

Next, both the potential noxious and beneficial effects of 24-OHC in AD pathogenesis are summarized.

### 4.1. Alzheimer’s Disease-Promoting Effects of 24-OHC

Numerous studies highlight the potential role of 24-OHC in favoring AD onset and progression.

Neuroinflammation plays a central role in AD pathogenesis since it might contribute to further neuronal dysfunction and cell death. Although astrocytes and microglia are the main players in neuroinflammation, it has been suggested that neurons may also contribute to chronic neuroinflammatory changes that occur in AD by releasing inflammatory mediators [103]. In this connection, 24-OHC has been shown to elicit a strong pro-inflammatory response in human neuroblastoma SH-SY5Y cells by inducing the expression of some pro-inflammatory mediators, including the chemokines interleukin 8 (IL-8) and monocyte chemotactic protein 1 (MCP-1), the adhesion molecule β1-integrin, the scavenger receptor CD36 and the matrix metalloproteinase 9 (MMP-9). This effect was found to occur via Toll-like receptor-4/cyclooxygenase-2/membrane bound prostaglandin E synthase (TLR4/COX-2/mPGES-1) [96]. Moreover, an oxysterol mixture representative of that found in the AD brain, in which 24-OHC is the main component (30–50%), induces a morphological change in mouse primary astrocytes, a clear indicator of astrocyte reactivity. This change was accompanied by the up-regulation of some reactive astrocyte markers and the release of pro-inflammatory molecules. Moreover, oxysterol-treated astrocytes have been shown to exert a synaptotoxic effect on mouse primary neurons, mainly mediated by lipocalin 2 release [104].

With regard to 24-OHC noxious effects, it has been demonstrated that at the physiological concentration of 1 µM, it is not neurotoxic per se, but it can potentiate both the apoptotic and the necrogenic effects exerted by the neurotoxic Aβ_42_ peptide. This peculiar behavior of 24-OHC has been confirmed on different neuronal cells, specifically on differentiated human neuroblastoma SK-N-BE and NT2 cells, and on human dental-pulp neuron-like cells. In particular, this effect is due to 24-OHC’s ability to amplify the availability of a multi-receptor complex composed of CD36, β1-integrin and CD47 on the cell surface, which enhances Aβ binding to neurons and its consequent intracellular accumulation [94,97]. Of interest, binding of Aβ to neuronal membranes facilitates the formation of amyloid oligomers and fibrils, which are responsible for a series of structural and functional cell changes leading to AD-related synaptic dysfunction [105,106]. The reason why 24-OHC, unlike other oxysterols, amplifies Aβ toxicity appears to be its marked pro-oxidant action on neuronal cells. It up-regulates the cell steady-state levels of ROS (mainly H_2_O_2_) through nicotinamide adenine dinucleotide phosphate (NADPH) oxidase activation, which results in derangement of the cell redox equilibrium. Moreover, 24-OHC-dependent potentiation of Aβ neurotoxicity was completely inhibited by incubation of cells with the antioxidants quercetin or genistein, confirming ROS involvement [94,97]. Notably, the prominent localization of CYP46A1 around amyloid plaques and in the amyloid core, with greater prevalence in the surrounding area [58,63], suggests that the presence of 24-OHC in the vicinity of amyloid plaques might enhance the adhesion of large amounts of Aβ to neurons amplifying Aβ neurotoxic action. However, there are still conflicting thoughts around the involvement of 24-OHC in APP processing and Aβ production.

24-OHC (10 µM) has been demonstrated to significantly up-regulate APP levels in human primary cultures of almost equal populations of neuronal and glial cells [107]. In addition, it has been shown in our laboratory that 1 µM 24-OHC is able to induce APP processing toward β-amyloidogenesis in neuronal differentiated SK-N-BE cells. It significantly enhances both expression and synthesis of APP and β-secretase (BACE1), the crucial enzyme involved in APP cleavage for Aβ production. Moreover, to confirm 24-OHC’s role in the pro-amyloidogenic pathway, BACE1 activation and Aβ increased levels were demonstrated, despite the fact that 24-OHC showed a parallel ability to up-regulate the protective enzyme α-secretase [93]. Conversely, the few other data available on the effect of 24-OHC on APP protein levels and β-amyloidogenesis either show no effect or a protective effect of this oxysterol [58,108,109].

With respect to direct cytotoxicity, 24-OHC has been shown to damage neuronal cells causing elevation in intracellular calcium and cell necrosis by increased generation of free radicals when added at a high concentration (50 µM) to undifferentiated neuroblastoma SH-SY5Y cells [110]. The same team later proved that 25 µM 24-OHC-induced neurotoxicity in differentiated SH-SY5Y cells, which are more vulnerable to 24-OHC than undifferentiated ones, is due to apoptosis and secondary necrosis, and might be partially prevented by vitamin E and estradiol-17β [111]. Moreover, it has been reported that relatively high concentrations of 24-OHC (10–50 µM) elicit caspase-independent programmed cell death, i.e., necroptosis in human neuroblastoma SH-SY5Y cells and in rat primary cortical neurons. In particular, 24-OHC-dependent necroptosis was dependent on the receptor-interacting serine/threonine kinase 1 (RIPK1) [95]. The same group further demonstrated that the initial key events in 24-OHC-induced necroptosis-like cell death were acyl-CoA:cholesterol acyltransferase 1 (ACAT1)-mediated esterification of 24-OHC and the resulting lipid droplet formation [112].

In addition to necroptosis, 24-OHC has been considered a potent inducer of oxiapoptophagy, a complex type of cell death involving simultaneous oxidative stress, apoptosis and autophagy. In fact, it has been demonstrated in 158N murine oligodendrocytes that, at the high concentration of 50 µM, this oxysterol inhibits cell proliferation, induces alteration of mitochondrial activity, overproduction of ROS and caspase 3 activation, and also causes other typical features of oxidative stress and apoptosis. Furthermore, 24-OHC promotes conversion of the microtubule-associated protein light chain 3 class I (LC3-I) to LC3-II, a specific marker of autophagy, supporting the idea that this oxysterol also has the ability to induce autophagy [113].

It has also been reported that 24-OHC can affect the renin-angiotensin system (RAS) in the brain. Besides the key systemic functions of RAS (e.g., regulation of blood pressure and electrolytic homeostasis), this system has been suggested to regulate several brain functions such as neuronal plasticity, learning, and memory [114]. 24-OHC (1 µM) has been shown to up-regulate several members of the brain RAS in primary neurons and astrocytes, partially through an LXR-dependent mechanism, including angiotensinogen (AGT), angiotensin-converting enzyme (ACE) and angiotensin II type 1 receptor (ATR1) [115]. Moreover, a significant increase in both ACE2 and Mas receptor expression was found in rat primary neurons treated with 1 µM 24-OHC [116]. Interestingly, high levels of AGT and ACE have been found in the CSF of patients with MCI and AD [117] and the over-activation of ATR1 has been shown to exacerbate cognitive impairment in 5XFAD AD mice [118] and contributes to Aβ production, oxidative stress and inflammation [119].

Another function of brain RAS is the modulation of glucose uptake by regulating glucose transporter-4 (GLUT4) vesicular trafficking [120]. The oxysterol 27-OHC has been shown to reduce brain glucose uptake and spatial memory in vivo by modulating brain RAS [121]. Although there is no evidence that 24-OHC-mediated RAS up-regulation affects brain glucose uptake, its ability to influence brain RAS similarly to 27-OHC [115] leads us to hypothesize that 24-OHC could impact this process. Overall, these data support the presence of a link between cholesterol metabolism, RAS and neurodegeneration [14].

### 4.2. Beneficial Effects of 24-OHC

As mentioned above, of all the oxysterols, 24-OHC is certainly the one with the most controversial role since it may act as either a pro-survival or a pro-death factor in neurons. These opposing effects may depend on 24-OHC levels since low concentrations seem to induce adaptive responses and beneficial effects. In support of this, it has been reported that certain lipid peroxidation products induce adaptive responses against subsequent further oxidative stress, thus preventing cell death [122,123,124]. In this connection, 24-OHC at sub-lethal concentrations (1–10 µM), up-regulates LXR target genes in neurons and generates a neuroprotective response by protecting the cells against subsequent cytotoxic stress and cell death induced by the oxysterol 7-KC. Among the LXR target genes, ABCG1 was demonstrated to be involved in the 24-OHC-induced adaptive response [100], probably by mediating intracellular and extracellular redistribution of 7-KC [125].

In AD, neurons expressing the enzyme choline acetyltransferase (ChAT) progressively degenerate leading to the loss of cholinergic activity that correlates with cognitive decline. Another important beneficial effect of 24-OHC is its ability to delay the decrease in ChAT-positive neurons in organotypic brain slices of the basal nucleus of Meynert [126].

It has been proposed that the neuroprotective action of 24-OHC may also involve allosteric modulation of NMDAR function, but in this case the activity of 24-OHC is the result of its direct binding to NMDARs and not of LXR activation [54,127]. In fact, it has been demonstrated that 24-OHC is a very potent, direct and selective positive allosteric modulator of NMDARs, a major subtype of glutamate receptors mediating excitatory transmission throughout the CNS, that have been shown to play critical roles in neuronal signaling and survival and, thus, in neuromodulatory functions. In particular, 24-OHC potentiates NMDAR-mediated excitatory postsynaptic currents and enhances long-term potentiation [54,128,129]. Due to this property, 24-OHC has been proposed to exert a crucial neuroprotective role for synaptic plasticity and learning. It has also been shown that 24-OHC (0.1–10 µM) is able to potentiate NMDAR-mediated responses and restore cognitive deficit in rodents treated with NMDAR channel blockers [54]. Specifically, 24-OHC was found to act mainly on NMDARs containing the GluN2B subunit in hippocampal neurons [130]. Notably, this subunit is an important target of memantine, the drug approved to ameliorate AD symptoms, which acts by inhibiting the extra-synaptic NMDARs, thus reducing glutamate excitotoxicity [131].

In addition, the ability of 24-OHC to exert protective effects against amyloid plaque formation has been described. The altered clearance of Aβ peptides that accumulate around brain microvessels of the BBB and in the brain parenchyma, together with NFT formation, promotes neuronal dysfunction, cell death and progressive cognitive decline [2]. Interestingly, 24-OHC has been shown to protect the brain from peripheral Aβ peptide entry. In fact, it decreases the influx of Aβ across brain microvessel ECs through the activation of LXRs and the consequent modulation of the expression of ABCB1, a transporter involved in the restriction of Aβ influx [43]. Moreover, with regard to Aβ production in the brain capillary ECs, 24-OHC has been demonstrated to inhibit the amyloidogenic cleavage of APP by reducing BACE1 expression and promoting the release of the soluble fragment sAPPα associated with the non-amyloidogenic pathway [132]. Moreover, in human neuroblastoma SH-SY5Y cells and CHO cells stably expressing human APP, 24-OHC has been shown to inhibit intracellular APP trafficking leading to immature APP retention in the endoplasmic reticulum (ER) without affecting secretase activities, while still suppressing Aβ production [99]. Moreover, it has been demonstrated that 24-OHC inhibits the secretion of Aβ by increasing APP processing via the non-amyloidogenic α-secretase pathway in rat primary neurons [58] and in SH-SY5Y neuroblastoma cells [109]. Another paper published in 2007 confirmed that 24-OHC favors the non-amyloidogenic APP cleavage by increasing the α-secretase activity as well as the α/β-secretase activity ratio [108].

Although much is known about the link between altered cholesterol metabolism and Aβ accumulation, its relationship with tau pathology is currently almost unknown, with few exceptions. Intraneuronal accumulation of NFTs made of hyperphosphorylated tau directly correlates with cognitive decline in AD and other primary tauopathies. Recently, we showed that 1 µM 24-OHC up-regulates both expression and synthesis of the neuroprotective enzyme sirtuin 1 (SIRT1) in neuroblastoma SK-N-BE cells, consequently preventing the intracellular accumulation of insoluble tau aggregates in neurons [98]. It has been hypothesized that 24-OHC favors tau degradation by inducing SIRT1-dependent deacetylation of tau. In this way, tau would become more susceptible to ubiquitination and proteasomal degradation, leading to total tau reduction in neurons [133]. Interestingly, the levels of SIRT1 markedly decrease in the brain with AD progression, in parallel with the loss of 24-OHC and accumulation of NFTs [57]. The ability of 24-OHC to induce SIRT1 synthesis and to prevent tau phosphorylation is supported by in vivo evidence obtained following the intra-cerebroventricular injection of 24-OHC in tau mice that develop tau pathology after Aβ monomer administration [98].

## 5. Therapeutic Approaches Targeting 24-OHC

Given that 24-OHC is a relevant mediator in AD etiology, one could speculate whether targeting this molecule would be therapeutically useful for disease prevention or could at least slow down its progression. In this regard, however, it is essential to establish what the purpose of the therapy should be, namely whether to counteract or promote 24-OHC production. Unfortunately, the literature is not yet able to give indication in this regard.

### 5.1. Effects of Statins on 24-OHC Levels

According to the view that hypercholesterolemia is included among the major risk factors for AD, several investigations focused on the possible application of statins in clinical practice. Besides their cholesterol lowering capability, some statins, in particular the lipophilic ones, might cross the BBB and exert anti-inflammatory and antioxidant effects within the CNS. Due to their pleiotropic action, they have recently been given more consideration for the care of AD [134,135,136,137,138].

Acting as inhibitors of 3-hydroxy-3-methylglutaryl CoA (HMG-CoA)-reductase—the key enzyme for cholesterol biosynthesis—it is reasonable to think that these drugs not only lower endogenous cholesterol synthesis but can also affect the amount of cholesterol metabolites, including 24-OHC, that in some cases have been demonstrated to exert neurotoxic effects.

Simvastatin, namely the most lipophilic statin, appears to be the major candidate for the regulation of 24-OHC, whose concentrations appear to decrease in the plasma of hypercholesterolemic AD patients, independently of total cholesterol reduction after 6 weeks of treatment with a high dose of the drug (80 mg/day). This supports the hypothesis that the drug could have other pharmacological activities besides the cholesterol-lowering property responsible for its effect on 24-OHC levels [139].

In another report, in AD patients treated with simvastatin (40 mg/day) both cholesterol and 24-OHC levels were found slightly but significantly reduced by about 11% and 9%, respectively [140]. Similarly, in AD patients with plasma total cholesterol levels higher than 160 mg/dL, administration of standard doses (40 mg/day) of simvastatin for 6 weeks lowered 24-OHC plasma levels, but 24-OHC/cholesterol ratio did not change and 24-OHC/LDL ratio markedly increased. Of note, even the less or not at all lipophilic statins lovastatin and pravastatin were able to reduce 24-OHC, accounting for their extra-cerebral mechanisms of action, such as LDL clearance from circulation [141].

High doses of simvastatin (80 mg/day) induced a significant decrease in 24-OHC levels in the CSF of normocholesterolemic patients with mild AD, in correlation with Aβ_40_ reduction, although cholesterol content was not affected. Such alterations were not observed in more severely affected patients [142].

Unfortunately, almost no studies report whether there exists a direct correlation between 24-OHC amounts in patients and clinical outcomes (e.g., memory, cognition and behavioral improvement) after statin administration. Indeed, as mentioned above, 24-OHC is usually evaluated as a marker to validate drug efficacy in regulating cholesterol homeostasis, being altered cholesterol homeostasis a risk factor for AD progression. Furthermore, the exact mechanisms of statins’ action in counteracting AD is not clarified, thus it cannot be ruled out whether these compounds behave as antioxidants, anti-inflammatory molecules and/or 24-OHC modulators.

### 5.2. Therapeutic Potential of Cholesterol 24-Hydroxylation by CYP46A1

Considering the emerging evidence supporting a positive role of 24-OHC in AD pathology, a potential pharmacological strategy for AD treatment could be acting directly on its endogenous production. This can be achieved by up-regulating the enzyme CYP46A1, whose levels are reduced in the AD brain [57,134,143].

The effect of 24-OHC modulation has been studied by affecting CYP46A1 in different in vitro and animal models, but not in humans.

The generation of CYP46A1 knock-out mice was the first approach to genetic manipulation of CYP46A1 activity in mammals [17]. Interestingly, the lack of CYP46A1 results in a great reduction of 24-OHC levels [144], but it does not affect the steady-state cholesterol levels nor the levels of other cholesterol metabolites. This suggests that cholesterol hydroxylation by CYP46A1 and the subsequent excretion of 24-OHC represent a robust tissue-specific pathway for cholesterol turnover in the brain [17,144]. In CYP46A1 knock-out animals with severe deficiencies in spatial, associative, motor learning and hippocampal long term potentiation have been observed [51]. Conversely, mice over-expressing CYP46A1 showed improved memory and hyper-activation of NMDARs [145].

It is also possible to modulate CYP46A1 activity at the gene level, for example by CYP46A1 ablation or by injection of an adeno-associated vector (AVV) encoding CYP46A1. To down-regulate CYP46A1 expression in the hippocampus of wild type mice, Djelti and colleagues used an AVV vector encoding short hairpin RNA directed against the mouse CYP46A1 gene. In this way, they showed that CYP46A1 inhibition led to cholesterol accumulation in neurons, Aβ production, abnormal tau phosphorylation, ER stress and apoptotic neuronal death, followed by hippocampal atrophy and memory impairment. Notably, these effects were stronger in the APP23 mouse model of AD [146].

The injection of AVV encoding CYP46A1 in the hippocampus of AD mice (APP23 or APP/PS mice) represents the first genetic manipulation to enhance CYP46A1 expression and activity in mammals. This injection, which increased CYP46A1 expression and 24-OHC levels in the brain, was able to reduce Aβ plaques and restore spatial memory performances [147]. In line with this, Burlot and colleagues demonstrated that bilateral hippocampal injections of AVV-CYP46A1 to THY Tau22 mice, a model of AD-like tau pathology where both CYP46A1 and 24-OHC levels are lower than normal, selectively enhanced CYP46A1 expression and restored 24-OHC levels in hippocampal neurons. Thanks to these injections, cognitive deficits, impaired long-term depression and spine defects that characterize these mice were completely rescued. Moreover, CYP46A1 over-expression rescued synaptic processes, dendritic length and spine density, but did not affect tau phosphorylation and related gliosis [148].

Another strategy to counteract AD progression via CYP46A1 might be its activation by the antiviral drug Efavirenz. Mast and colleagues demonstrated that the treatment of 5XFAD mice, a model of rapid amyloidogenesis, with a low dose of Efavirenz led to the enhancement of CYP46A1 activity, reduced amyloid burden and microglia activation in the cerebral cortex and subiculum, and rescued spatial and non-spatial memory [149]. Other compounds including endogenous neuroactive molecules, have been tested in vitro with purified recombinant CYP46A1. Among these, L-glutamine was shown to elicit the highest increase in CYP46A1-mediated cholesterol 24-hydroxylation. Moreover, L-glutamine and Efavirenz synergistically activate CYP46A1 [150].

As recently demonstrated in our laboratory, besides CYP46A1 targeting approaches, the direct intra-cerebroventricular injection of 24-OHC could counteract the accumulation of hyperphosphorylated tau by activation of the SIRT1 neuroprotective pathway [98].

## 6. Conclusions

**Figure 2:**
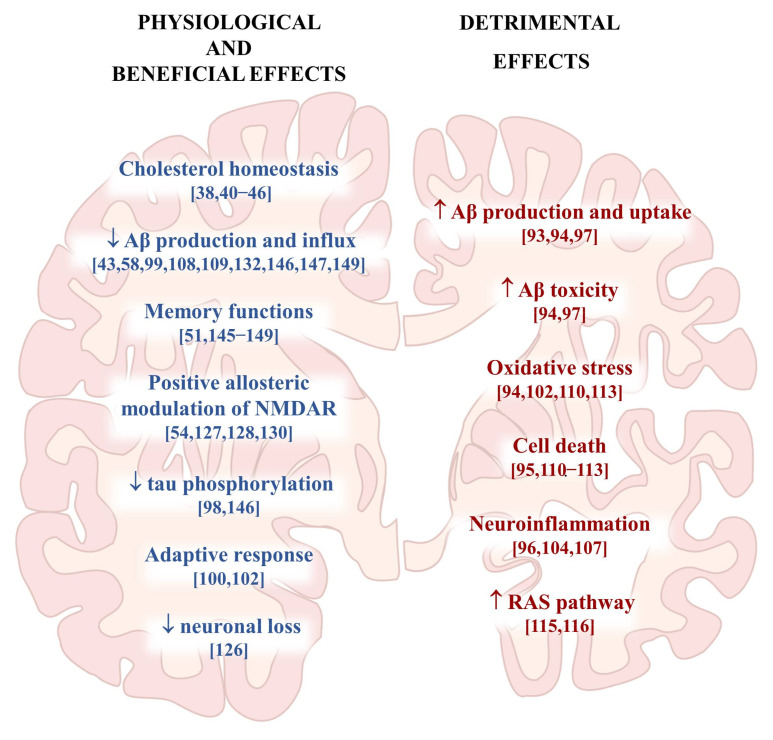
Representation of the most relevant effects of 24-S-hydroxycholesterol in the brain.

There are many data concerning the role of 24-OHC in the pathogenesis of AD, indicating that it can act as a double-edged sword, as depicted in Figure 2. Due to its dual role, it is often difficult to understand and interpret the data as a whole. For this reason, this is still an active area of research. Despite these conflicting data, one can assume that the physiological presence of this oxysterol in the brain is fundamental to guarantee brain health, as highlighted by the modulation of CYP46A1 activity in vivo. This suggests the importance of preventing the loss of 24-OHC in the brain during the course of AD. Overall, the results obtained to date concerning an attempt to prevent the loss of cerebral 24-OHC appear interesting, but more in-depth investigations are needed to further elucidate the reliability and feasibility of CYP46A1-targeting approaches, in particular when considering its application in humans.

## Data Availability

Data is contained within the article.
